# Effectiveness of Artificial Neural Networks for Solving Inverse Problems in Magnetic Field-Based Localization

**DOI:** 10.3390/s22062240

**Published:** 2022-03-14

**Authors:** Ai-ichiro Sasaki

**Affiliations:** Department of Electronic Engineering and Computer Science, Kindai University, Higashi-Hiroshima 739-2116, Japan; aisasaki@hiro.kindai.ac.jp; Tel.: +81-82-439-1110 (ext. 620)

**Keywords:** artificial neural networks, indoor localization, inverse problem, *k*-nearest neighbor algorithm, magnetic field, optimization, real-time tracking

## Abstract

Recently, indoor localization has become an active area of research. Although there are various approaches to indoor localization, methods that utilize artificially generated magnetic fields from a target device are considered to be the best in terms of localization accuracy under non-line-of-sight conditions. In magnetic field-based localization, the target position must be calculated based on the magnetic field information detected by multiple sensors. The calculation process is equivalent to solving a nonlinear inverse problem. Recently, a machine-learning approach has been proposed to solve the inverse problem. Reportedly, adopting the *k*-nearest neighbor algorithm (*k*-NN) enabled the machine-learning approach to achieve fairly good performance in terms of both localization accuracy and computational speed. Moreover, it has been suggested that the localization accuracy can be further improved by adopting artificial neural networks (ANNs) instead of *k*-NN. However, the effectiveness of ANNs has not yet been demonstrated. In this study, we thoroughly investigated the effectiveness of ANNs for solving the inverse problem of magnetic field-based localization in comparison with *k*-NN. We demonstrate that despite taking longer to train, ANNs are superior to *k*-NN in terms of localization accuracy. The *k*-NN is still valid for predicting fairly accurate target positions within limited training times.

## 1. Introduction

Location-based services have become indispensable in daily life. This is primarily owing to global positioning systems (GPS), whose performance has reached an unprecedentedly high level [1]. The significance of location-based services will increase in a full-blown era of the Internet of Things (IoT) [2]. Although GPS effectively localizes objects existing outdoors, it is not suitable for indoor localization since radio-wave propagation is disturbed by buildings. Indoor localization techniques have been actively studied to compensate for the limitations of GPS [3,4,5,6,7].

There are two main approaches for realizing indoor localization. One approach uses radio waves [5,6,7]. Since radio waves can reach far points, it is possible with this approach to obtain a large coverage area. A remarkable feature of radio waves is that they are reflected by walls, floors, and obstacles. On account of this feature, it has been a severe problem to improve localization accuracy with radio waves.

The second approach uses magnetic fields. The magnetic field-based approach is further divided into two different methods. One method utilizes geomagnetic fields, which are static fields [8,9,10,11]. The geomagnetic field-based method adopts fingerprinting techniques and is conducive for covering relatively wide areas. To execute the geomagnetic field-based localization, we must gather considerable amounts of data of the field strength at various points of the target area in advance. Moreover, it is required to update the magnetic field data since geomagnetic field patterns gradually change over periods of time. Since it is a tedious task to frequently gather the numerous data, establishing accurate localization systems using geomagnetic fields is very tough.

The second magnetic field-based localization method utilizes artificially generated magnetic fields, which are not static but time-varying fields [12,13,14,15]. Generally, the frequencies used for this method are within low-frequency (LF) or high-frequency (HF) bands. A remarkable feature of artificially generated magnetic fields is that the field patterns can be easily calculated with reasonable precision. Owing to this feature, the tedious task involved in the geomagnetic field-based method is no longer needed with the artificially generated fields. The other feature of magnetic fields is that the interaction between the fields and materials is much weaker than that between radio waves and materials, and this feature is not only limited to artificially generated fields but also to geomagnetic fields. Owing to these two features, the method involving artificially generated magnetic fields is best in terms of localization accuracy in non-line-of-sight (NLOS) environments [4].

Localization accuracy is considered the most important aspect for location-based services. Therefore, in this study, we treat localization techniques based on artificially generated magnetic fields. The only weakness of these techniques is that their coverage distance is typically limited to less than 10 m, which is much shorter than that of radio waves. It is due to the fact that artificially generated magnetic fields attenuate more steeply than radio waves. Localization techniques that combine radio waves with a magnetic field-based approach have been proposed to compensate for the weak point of artificially generated fields [5,6]. Moreover, techniques for amplifying magnetic fields without increasing the power consumption have also been investigated [16,17].

Typical localization systems that use artificially generated magnetic fields are composed of multiple sensors placed at various positions within the target area. Magnetic fields generated by a target device are detected by these sensors. The position of the target device must be calculated using the magnetic field data detected by the multiple sensors. However, this calculation is not trivial since it involves solving nonlinear inverse problems. Three different methods are known for solving inverse problems in localization with artificially generated magnetic fields.

The simplest method entails directly calculating the position of the target using closed-form formulae [12]. The main advantage of this method is its short computational time. It becomes possible with this method to establish real time location tracking of persons and objects. However, the method is applicable only to systems having limited sensor configurations in terms of numbers, positions, and their angles. Therefore, it is difficult to flexibly design localization systems with the simplest method.

The second method solves optimization problems that are reduced from inverse problems. The conventional optimization method can be employed to predict the position of the target from the detected signals by minimizing an appropriately constructed objective function [3,13,14]. An advantage of the optimization method is that the method can be utilized for any sensor configurations. Therefore, the optimization method surpasses the closed-form formulae method from a viewpoint of flexibility in the system design. However, it is difficult to implement real-time tracking of moving targets with the optimization method since it requires a much longer computational time. Moreover, since the optimization method requires certain skills, it is not easy for beginners to obtain appropriate solutions using the method. In fact, experienced engineers are often bothered about the problem of local minima.

The third method involves the application of machine learning to solve the inverse problems [18,19]. We have demonstrated that the computational time required for calculating the target position is significantly reduced by adopting machine learning. Moreover, it has been shown that the prediction accuracy of the target position is improved by using machine learning instead of the conventional optimization method. Therefore, it is expected that real-time tracking of moving targets is possible with high precision using machine learning. Additionally, machine learning can be applied to any sensor configuration, which is a significant advantage. Furthermore, even beginners can easily obtain appropriate solutions for inverse problems as many useful machine-learning tools are becoming increasingly available.

Since there are many algorithms for machine learning, it is critical to select an algorithm suitable for magnetic field-based localization. It has been demonstrated that we can predict the target positions in reasonable accuracy within a short computational time by adopting the *k*-nearest neighbor algorithm (*k*-NN). Furthermore, it is well known that artificial neural networks (ANNs) exhibit excellent performance in various machine-learning applications. It was also suggested that better prediction accuracy may be obtained with ANNs in comparison with *k*-NN [20]. However, a performance comparison between these two algorithms has not yet been reported.

In this study, we thoroughly investigated the effectiveness of ANNs for magnetic field-based localization in comparison with *k*-NN. In Section 2, we formulate the inverse problems to be solved for realizing magnetic field-based localization. Moreover, the machine-learning approach for solving inverse problems is also explained. In Section 3, the performances obtained with the two different machine-learning algorithms, ANNs and *k*-NN, are compared from various viewpoints. It was demonstrated that ANNs exhibit better prediction accuracy, although they require a much longer training time in comparison with *k*-NN.

## 2. Formulation of Inverse Problems

In this section, we describe the basic of calculation techniques required for magnetic field-based localization and formulate the inverse problems to be solved by machine learning [18,19].

### 2.1. Calculation of Artificially Generated Magnetic Fields

**Figure 1:**
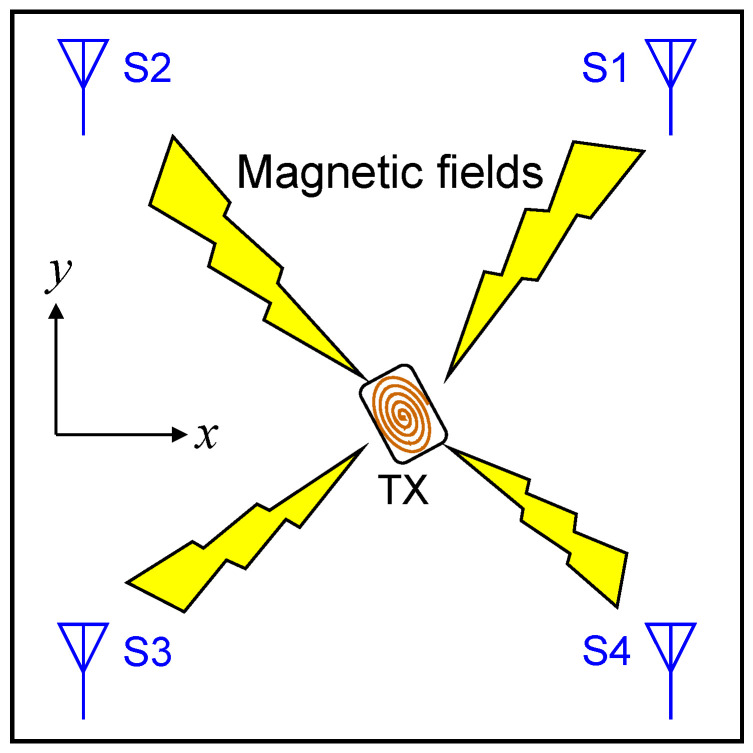
A conceptual image of a localization system with artificially generated magnetic fields (Top view). Magnetic fields generated by a TX are detected by multiple sensors (S1–S4) located at various points of the target space. The position of the TX is calculated from the information of the magnetic fields detected by the sensors.

**Figure 2:**
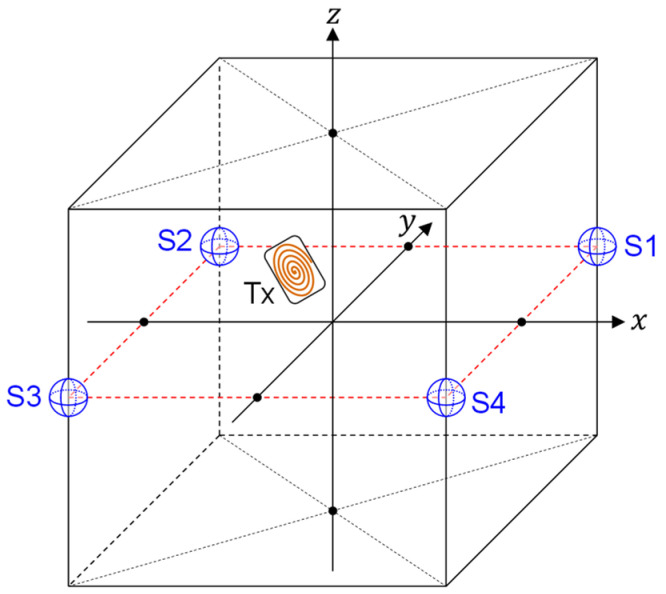
A three-dimensional (3D) drawing of a localization system with artificially generated magnetic fields.

Figure 1 and Figure 2 show a typical localization system that utilizes artificially generated magnetic fields. Suppose that the target device having a transmitter (TX) is freely moving inside the cubic space. Magnetic fields are generated from a single coil connected to the TX and they are detected by magnetic field sensors (S1–S4) placed at four corners. Although the four sensors are depicted in Figure 1 and Figure 2 as an example, the number of sensors is not limited to four. Furthermore, locations of the sensors are not limited to the corners. Our machine-learning approach has no limitation regarding the number and location of the sensors. In this study, however, we assume that four sensors are located at four corners of the cubic space.

We also consider that the sensors can detect all components of the magnetic field vectors $B=\left(B_{x},B_{y},B_{z}\right)$. In other words, each sensor is equipped with three receiving coils, that are perpendicular to each other. Our objective is to calculate the position of the TX based on the magnetic field information detected by the four sensors.

Magnetic field patterns generated inside the cubic space depend on both the position and angles of the TX coil. Therefore, magnetic-field vector components detected by the *k*-th sensor can be formally written as follows:

(1) $$B^{\left(k\right)}=\left(\begin{array}{c} B_{x}^{\left(k\right)}\\ B_{y}^{\left(k\right)}\\ B_{z}^{\left(k\right)} \end{array}\right)=\left(\begin{array}{c} B_{x}^{\left(k\right)}\left(x^{\left(t\right)},y^{\left(t\right)},z^{\left(t\right)},\theta^{\left(t\right)},\varphi^{\left(t\right)}\right)\\ B_{y}^{\left(k\right)}\left(x^{\left(t\right)},y^{\left(t\right)},z^{\left(t\right)},\theta^{\left(t\right)},\varphi^{\left(t\right)}\right)\\ B_{z}^{\left(k\right)}\left(x^{\left(t\right)},y^{\left(t\right)},z^{\left(t\right)},\theta^{\left(t\right)},\varphi^{\left(t\right)}\right) \end{array}\right),$$

where $\left(x^{\left(t\right)},y^{\left(t\right)},z^{\left(t\right)}\right)$ denote the position of the TX coil and $\left(\theta^{\left(t\right)},\varphi^{\left(t\right)}\right)$ represent the direction of a vector normal to the TX coil in spherical coordinates. The superscript “(t)” indicates that the symbols having “(t)” represent the physical quantities of the TX. Hereinafter, $\left(x^{\left(t\right)},y^{\left(t\right)},z^{\left(t\right)},\theta^{\left(t\right)},\varphi^{\left(t\right)}\right)$ are referred to as “TX-state parameters” since they represent the physical state of the TX.

The magnetic fields created at an arbitrary point (x,y,z) by the TX coil placed at a coordinate origin can be calculated using the following equation [18,19]:

(2) $$\left(\begin{array}{c} B_{x}\left(x,y,z,\theta^{\left(t\right)},\varphi^{\left(t\right)}\right)\\ B_{y}\left(x,y,z,\theta^{\left(t\right)},\varphi^{\left(t\right)}\right)\\ B_{z}\left(x,y,z,\theta^{\left(t\right)},\varphi^{\left(t\right)}\right) \end{array}\right)=\frac{\mu_{0}}{4\pi }\frac{m}{{\left(x^{2}+y^{2}+z^{2}\right)}^{5/2}}\times [\cos\theta^{\left(t\right)}\left(\begin{array}{c} 3xz\\ 3yz\\ 2z^{2}-x^{2}-y^{2} \end{array}\right)\;+\sin\theta^{\left(t\right)}\left(\begin{array}{c} 3xy\sin\varphi^{\left(t\right)}+\left(2x^{2}-y^{2}-z^{2}\right)\cos\varphi^{\left(t\right)}\\ 3xy\cos\varphi^{\left(t\right)}+\left(2x^{2}-y^{2}-z^{2}\right)\sin\varphi^{\left(t\right)}\\ 3z\left(x\cos\varphi^{\left(t\right)}+y\sin\varphi^{\left(t\right)}\right) \end{array}\right)],$$

where $\mu_{0}$ and m denote the vacuum permeability and dipole moment associated with the TX coil, respectively. The dipole moment can be expressed by the parameters of an *N*-turn coil attached to the TX as follows:

(3) m=NIS,

where I and S denote the current and area size of the TX coil, respectively.

Note that the magnetic-field vector components detected by the *k*-th sensor can be written in terms of the left-hand side of Equation (2) as follows:

(4) $$\left(\begin{array}{c} B_{x}^{\left(k\right)}\left(x^{\left(t\right)},y^{\left(t\right)},z^{\left(t\right)},\theta^{\left(t\right)},\varphi^{\left(t\right)}\right)\\ B_{y}^{\left(k\right)}\left(x^{\left(t\right)},y^{\left(t\right)},z^{\left(t\right)},\theta^{\left(t\right)},\varphi^{\left(t\right)}\right)\\ B_{z}^{\left(k\right)}\left(x^{\left(t\right)},y^{\left(t\right)},z^{\left(t\right)},\theta^{\left(t\right)},\varphi^{\left(t\right)}\right) \end{array}\right)=\left(\begin{array}{c} B_{x}\left(x^{\left(k\right)}-x^{\left(t\right)},y^{\left(k\right)}-y^{\left(t\right)},z^{\left(k\right)}-z^{\left(t\right)},\theta^{\left(t\right)},\varphi^{\left(t\right)}\right)\\ B_{y}\left(x^{\left(k\right)}-x^{\left(t\right)},y^{\left(k\right)}-y^{\left(t\right)},z^{\left(k\right)}-z^{\left(t\right)},\theta^{\left(t\right)},\varphi^{\left(t\right)}\right)\\ B_{z}\left(x^{\left(k\right)}-x^{\left(t\right)},y^{\left(k\right)}-y^{\left(t\right)},z^{\left(k\right)}-z^{\left(t\right)},\theta^{\left(t\right)},\varphi^{\left(t\right)}\right) \end{array}\right),$$

where $\left(x^{\left(k\right)},y^{\left(k\right)},z^{\left(k\right)}\right)$ denote the positions of the *k*-th sensor. Substituting Equation (2) into the right-hand side of Equation (4), we can derive a useful formula to calculate the magnetic-field vector components detected by the *k*-th sensor for any TX-state parameters as shown below.

(5) $$\left(\begin{array}{c} B_{x}^{\left(k\right)}\left(x^{\left(t\right)},y^{\left(t\right)},z^{\left(t\right)},\theta^{\left(t\right)},\varphi^{\left(t\right)}\right)\\ B_{y}^{\left(k\right)}\left(x^{\left(t\right)},y^{\left(t\right)},z^{\left(t\right)},\theta^{\left(t\right)},\varphi^{\left(t\right)}\right)\\ B_{z}^{\left(k\right)}\left(x^{\left(t\right)},y^{\left(t\right)},z^{\left(t\right)},\theta^{\left(t\right)},\varphi^{\left(t\right)}\right) \end{array}\right)=\frac{\mu_{0}}{4\pi }\frac{m}{{\left\{{\left(x^{\left(k\right)}-x^{\left(t\right)}\right)}^{2}+{\left(y^{\left(k\right)}-y^{\left(t\right)}\right)}^{2}+{\left(z^{\left(k\right)}-z^{\left(t\right)}\right)}^{2}\right\}}^{5/2}}\times \;[\cos\theta^{\left(t\right)}\left(\begin{array}{c} 3\left(x^{\left(k\right)}-x^{\left(t\right)}\right)\left(z^{\left(k\right)}-z^{\left(t\right)}\right)\\ 3\left(y^{\left(k\right)}-y^{\left(t\right)}\right)\left(z^{\left(k\right)}-z^{\left(t\right)}\right)\\ 2{\left(z^{\left(k\right)}-z^{\left(t\right)}\right)}^{2}-{\left(x^{\left(k\right)}-x^{\left(t\right)}\right)}^{2}-{\left(y^{\left(k\right)}-y^{\left(t\right)}\right)}^{2} \end{array}\right)\;+\sin\theta^{\left(t\right)}\left(\begin{array}{c} 3\left(x^{\left(k\right)}-x^{\left(t\right)}\right)\left(y^{\left(k\right)}-y^{\left(t\right)}\right)\sin\varphi^{\left(t\right)}+\left\{2{\left(x^{\left(k\right)}-x^{\left(t\right)}\right)}^{2}-{\left(y^{\left(k\right)}-y^{\left(t\right)}\right)}^{2}-{\left(z^{\left(k\right)}-z^{\left(t\right)}\right)}^{2}\right\}\cos\varphi^{\left(t\right)}\\ 3\left(x^{\left(k\right)}-x^{\left(t\right)}\right)\left(y^{\left(k\right)}-y^{\left(t\right)}\right)\cos\varphi^{\left(t\right)}+\left\{2{\left(x^{\left(k\right)}-x^{\left(t\right)}\right)}^{2}-{\left(y^{\left(k\right)}-y^{\left(t\right)}\right)}^{2}-{\left(z^{\left(k\right)}-z^{\left(t\right)}\right)}^{2}\right\}\sin\varphi^{\left(t\right)}\\ 3\left(z^{\left(k\right)}-z^{\left(t\right)}\right)\;\left\{\left(x^{\left(k\right)}-x^{\left(t\right)}\right)\cos\varphi^{\left(t\right)}+\left(y^{\left(k\right)}-y^{\left(t\right)}\right)\sin\varphi^{\left(t\right)}\right\}\; \end{array}\right)]$$

### 2.2. Solving Inverse Problems Using Machine Learning

Since the above formula has been obtained, it is easy to calculate $\left(B_{x}^{\left(k\right)},B_{y}^{\left(k\right)},B_{z}^{\left(k\right)}\right)$ from the arbitrary TX-state parameters $\left(x^{\left(t\right)},y^{\left(t\right)},z^{\left(t\right)},\theta^{\left(t\right)},\varphi^{\left(t\right)}\right)$. It is a straightforward problem. However, magnetic field-based localization requires the calculation of $\left(x^{\left(t\right)},y^{\left(t\right)},z^{\left(t\right)}\right)$ from $\left(B_{x}^{\left(k\right)},B_{y}^{\left(k\right)},B_{z}^{\left(k\right)}\right)$, and the calculation is not easy since it is a nonlinear inverse problem. Solving the inverse problem is equivalent to finding a function F that satisfies

(6) $$x^{\left(t\right)}=F\left(B^{\left(1\right)},B^{\left(2\right)},B^{\left(3\right)},B^{\left(4\right)}\right),$$

where $x^{\left(t\right)}=\left(x^{\left(t\right)},y^{\left(t\right)},z^{\left(t\right)}\right)$. After observing Equation (5), it is hopeless to obtain the exact form of F. Therefore, the next step involves constructing a function that can “predict” the approximate values of the TX position based on the magnetic field information detected by the four sensors. In equation form, the predictor function P can be expressed as follows:

(7) $$x^{\left(p\right)}=P\left(B^{\left(1\right)},B^{\left(2\right)},B^{\left(3\right)},B^{\left(4\right)}\right),$$

where $x^{\left(p\right)}=\left(x^{\left(p\right)},y^{\left(p\right)},z^{\left(p\right)}\right)$ denotes predicted TX-position vector. It is emphasized with the superscript (p) that $x^{\left(p\right)}$ is the predicted quantity and is not identical to $x^{\left(t\right)}$, which is a true TX-position vector. Although $x^{\left(p\right)}$ is considered an approximation of $x^{\left(t\right)}$, we should note that $x^{\left(p\right)}$ is not completely equal to $x^{\left(t\right)}$. In the component representation, Equation (7) can be written as follows:

(8) $$\left(\begin{array}{c} x^{\left(p\right)}\\ y^{\left(p\right)}\\ z^{\left(p\right)} \end{array}\right)=(\begin{array}{c} P_{x}\left(B_{x}^{\left(1\right)},B_{y}^{\left(1\right)},B_{z}^{\left(1\right)},B_{x}^{\left(2\right)},B_{y}^{\left(2\right)},B_{z}^{\left(2\right)},B_{x}^{\left(3\right)},B_{y}^{\left(3\right)},B_{z}^{\left(3\right)},B_{x}^{\left(4\right)},B_{y}^{\left(4\right)},B_{z}^{\left(4\right)}\right)\\ P_{y}\left(B_{x}^{\left(1\right)},B_{y}^{\left(1\right)},B_{z}^{\left(1\right)},B_{x}^{\left(2\right)},B_{y}^{\left(2\right)},B_{z}^{\left(2\right)},B_{x}^{\left(3\right)},B_{y}^{\left(3\right)},B_{z}^{\left(3\right)},B_{x}^{\left(4\right)},B_{y}^{\left(4\right)},B_{z}^{\left(4\right)}\right)\\ P_{z}\left(B_{x}^{\left(1\right)},B_{y}^{\left(1\right)},B_{z}^{\left(1\right)},B_{x}^{\left(2\right)},B_{y}^{\left(2\right)},B_{z}^{\left(2\right)},B_{x}^{\left(3\right)},B_{y}^{\left(3\right)},B_{z}^{\left(3\right)},B_{x}^{\left(4\right)},B_{y}^{\left(4\right)},B_{z}^{\left(4\right)}\right) \end{array})$$

Generally, regression analysis is an effective method for constructing P. It is possible to construct P with reasonable accuracy through regression analysis by gathering sufficient volume of training data. In equation form, the training data are expressed as follows:

(9) $$\left(B^{\left(1\right)},B^{\left(2\right)},B^{\left(3\right)},B^{\left(4\right)}\right)⟼x^{\left(t\right)},$$

where the left- and right-hand sides denote the input and desired outputs of P, respectively. In the component representation, Equation (9) can be written as follows:

(10) $$\left(B_{x}^{\left(1\right)},B_{y}^{\left(1\right)},B_{z}^{\left(1\right)},B_{x}^{\left(2\right)},B_{y}^{\left(2\right)},B_{z}^{\left(2\right)},B_{x}^{\left(3\right)},B_{y}^{\left(3\right)},B_{z}^{\left(3\right)},B_{x}^{\left(4\right)},B_{y}^{\left(4\right)},B_{z}^{\left(4\right)}\right)\mapsto \left(x^{\left(t\right)},y^{\left(t\right)},z^{\left(t\right)}\right)$$

Hereinafter, the training-data representation used in Equations (9) and (10) is referred to as “linear representation”.

Generally speaking, it is often required to gather training data from a real world for the regression analysis. However, for our purpose, measurements in the real world are not required since the magnetic-field patterns formed by the TX coil can be calculated by Equation (2) in reasonable accuracy. Actually, existing localization systems that use artificially generated magnetic fields are based on the fact that the fields are governed by Equation (2) [12,13,14].

Therefore, for our purpose, sufficient quantity of training data can be easily generated by applying Equation (5) to various TX-state parameters. This is a straightforward procedure. Presently, it is well known that machine learning is effective for regression analysis. When machine learning works properly with a sufficient number of training data, we can obtain a reasonable predictor function P that satisfies Equations (7) and (8).

Given that $B^{\left(k\right)}$ depends on $x^{\left(t\right)}$, $\theta^{\left(t\right)}$, and $\varphi^{\left(t\right)}$, the predictor function can also be viewed as a function of these variables. Subsequently, we introduce a new predictor function, $\tilde{P}$ defined as

(11) $$x^{\left(p\right)}=P\left(B^{\left(1\right)}\left(x^{\left(t\right)},\theta^{\left(t\right)},\varphi^{\left(t\right)}\right),B^{\left(2\right)}\left(x^{\left(t\right)},\theta^{\left(t\right)},\varphi^{\left(t\right)}\right),B^{\left(3\right)}\left(x^{\left(t\right)},\theta^{\left(t\right)},\varphi^{\left(t\right)}\right),B^{\left(4\right)}\left(x^{\left(t\right)},\theta^{\left(t\right)},\varphi^{\left(t\right)}\right)\right)\;≜\tilde{P}\left(x^{\left(t\right)},\theta^{\left(t\right)},\varphi^{\left(t\right)}\right).$$

It is reasonable to appraise the performance of the predictor function by the difference between $x^{\left(t\right)}$ and $x^{\left(p\right)}$. Hence, we define an error distance function (EDF) as follows:

(12) $$d\left(x^{\left(t\right)},\theta^{\left(t\right)},\varphi^{\left(t\right)}\right)≜\|x^{\left(t\right)}-\tilde{P}{\left(x^{\left(t\right)},\theta^{\left(t\right)},\varphi^{\left(t\right)}\right)\|}_{2},$$

where the subscript “2” in the right-hand side of Equation (12) denotes that the EDF is defined by the Euclidean distance between $x^{\left(t\right)}$ and $x^{\left(p\right)}$. Since the EDF represents the error of the predictor function, the values of d decrease with improvements in the predictor functions.

### 2.3. Method for Generating Better Predictor Functions

When $\theta^{\left(t\right)}=0$, Equation (2) can be simplified by using a spherical coordinate.

(13) $$\left(\begin{array}{c} B_{r}\left(r,\theta ,\varphi \right)\\ B_{\theta }\left(r,\theta ,\varphi \right)\\ B_{\varphi }\left(r,\theta ,\varphi \right) \end{array}\right)=\frac{\mu_{0}}{4\pi }\frac{m}{r^{3}}\left(\begin{array}{c} 2\cos\theta \\ \sin\theta \\ 0 \end{array}\right)$$

Here (r,θ,φ) denote variables of the spherical coordinate system. Note that (θ,φ) are different from TX-state parameters $\left(\theta^{\left(t\right)},\varphi^{\left(t\right)}\right)$. It is understood from Equation (13) that the amplitude of fields generated by a magnetic source is proportional to $r^{-3}$. This is a remarkable property of near fields generated by a magnetic dipole, which is nothing but a current loop. In contrast, it is well known that the amplitude of radio waves is proportional to $r^{-1}$. More specifically, the spatial attenuation ratio of magnetic fields (60 dB/decade) is much larger than that of radio waves (20 dB/decade).

In this study, we treat the magnetic near fields, whose attenuation ratio is 60 dB/decade. Performances of the localization systems that use artificially generated magnetic fields are governed mainly by the large attenuation ratio. When the TX moves proximity of a sensor, the sensor detects a considerably larger signal level. Moreover, the variations in the detected signal level also become very large. On the other hand, when the TX are existing in the regions distant from a sensor, both the signal level and its variations become extremely small. It was suggested that the prediction accuracy obtained with machine learning is limited by these differences in the behavior of signal levels [18].

To solve this problem, an effective method for preprocessing training data has been proposed [19]. The core idea of the method is to logarithmically transform the magnetic field values in the training data. In the equation form, the transformation is written as follows:

(14) $${\hat{B}}_{i}^{\left(k\right)}≜\mathrm{sign}\left(B_{i}^{\left(k\right)}\right)\log\left(\frac{\left|B_{i}^{\left(k\right)}\right|}{\underset{\left(\mathrm{all}\;\mathrm{training}\;\mathrm{data}\right)}{\max}\left\{\left|B_{i}^{\left(k\right)}\right|\right\}}\right),$$

where i can be x, y, or z. Note that the positive/negative information of $B_{i}^{\left(k\right)}$ is encoded in ${\hat{B}}_{i}^{\left(k\right)}$. Moreover, a one-to-one correspondence is satisfied between $B_{i}^{\left(k\right)}$ and ${\hat{B}}_{i}^{\left(k\right)}$ [19]. The preprocessed training data are then written as follows:

(15) $$\left({\hat{B}}_{x}^{\left(1\right)},{\hat{B}}_{y}^{\left(1\right)},{\hat{B}}_{z}^{\left(1\right)},{\hat{B}}_{x}^{\left(2\right)},{\hat{B}}_{y}^{\left(2\right)},{\hat{B}}_{z}^{\left(2\right)},{\hat{B}}_{x}^{\left(3\right)},{\hat{B}}_{y}^{\left(3\right)},{\hat{B}}_{z}^{\left(3\right)},{\hat{B}}_{x}^{\left(4\right)},{\hat{B}}_{y}^{\left(4\right)},{\hat{B}}_{z}^{\left(4\right)}\right)⟼\left(x^{\left(t\right)},y^{\left(t\right)},z^{\left(t\right)}\right)$$

As with Equation (9), we can express Equation (15) in a simple form using vector notation.

(16) $$\left({\hat{B}}^{\left(1\right)},{\hat{B}}^{\left(2\right)},{\hat{B}}^{\left(3\right)},{\hat{B}}^{\left(4\right)}\right)⟼x^{\left(t\right)}$$

Hereinafter, the training-data representation used in Equations (15) and (16) is referred to as “normalized-signed log (NSL) representation.” Accordingly, we denote the predictor function generated by the training data in the NSL representation by Q. Subsequently, the equations corresponding to Equations (7), (8), (11), and (12) can be written as follows:

(17) $$x^{\left(p\right)}=Q\left({\hat{B}}^{\left(1\right)},{\hat{B}}^{\left(2\right)},{\hat{B}}^{\left(3\right)},{\hat{B}}^{\left(4\right)}\right)$$

(18) $$\left(\begin{array}{c} x^{\left(p\right)}\\ y^{\left(p\right)}\\ z^{\left(p\right)} \end{array}\right)=(\begin{array}{c} Q_{x}\left({\hat{B}}_{x}^{\left(1\right)},{\hat{B}}_{y}^{\left(1\right)},{\hat{B}}_{z}^{\left(1\right)},{\hat{B}}_{x}^{\left(2\right)},{\hat{B}}_{y}^{\left(2\right)},{\hat{B}}_{z}^{\left(2\right)},{\hat{B}}_{x}^{\left(3\right)},{\hat{B}}_{y}^{\left(3\right)},{\hat{B}}_{z}^{\left(3\right)},{\hat{B}}_{x}^{\left(4\right)},{\hat{B}}_{y}^{\left(4\right)},{\hat{B}}_{z}^{\left(4\right)}\right)\\ Q_{z}\left({\hat{B}}_{x}^{\left(1\right)},{\hat{B}}_{y}^{\left(1\right)},{\hat{B}}_{z}^{\left(1\right)},{\hat{B}}_{x}^{\left(2\right)},{\hat{B}}_{y}^{\left(2\right)},{\hat{B}}_{z}^{\left(2\right)},{\hat{B}}_{x}^{\left(3\right)},{\hat{B}}_{y}^{\left(3\right)},{\hat{B}}_{z}^{\left(3\right)},{\hat{B}}_{x}^{\left(4\right)},{\hat{B}}_{y}^{\left(4\right)},{\hat{B}}_{z}^{\left(4\right)}\right)\\ Q_{x}\left({\hat{B}}_{x}^{\left(1\right)},{\hat{B}}_{y}^{\left(1\right)},{\hat{B}}_{z}^{\left(1\right)},{\hat{B}}_{x}^{\left(2\right)},{\hat{B}}_{y}^{\left(2\right)},{\hat{B}}_{z}^{\left(2\right)},{\hat{B}}_{x}^{\left(3\right)},{\hat{B}}_{y}^{\left(3\right)},{\hat{B}}_{z}^{\left(3\right)},{\hat{B}}_{x}^{\left(4\right)},{\hat{B}}_{y}^{\left(4\right)},{\hat{B}}_{z}^{\left(4\right)}\right) \end{array})$$

(19) $$x^{\left(p\right)}=Q\left({\hat{B}}^{\left(1\right)}\left(x^{\left(t\right)},\theta^{\left(t\right)},\varphi^{\left(t\right)}\right),{\hat{B}}^{\left(2\right)}\left(x^{\left(t\right)},\theta^{\left(t\right)},\varphi^{\left(t\right)}\right),{\hat{B}}^{\left(3\right)}\left(x^{\left(t\right)},\theta^{\left(t\right)},\varphi^{\left(t\right)}\right),{\hat{B}}^{\left(4\right)}\left(x^{\left(t\right)},\theta^{\left(t\right)},\varphi^{\left(t\right)}\right)\right)\;≜\tilde{Q}\left(x^{\left(t\right)},\theta^{\left(t\right)},\varphi^{\left(t\right)}\right)$$

(20) $$d\left(x^{\left(t\right)},\theta^{\left(t\right)},\varphi^{\left(t\right)}\right)≜\|x^{\left(t\right)}-\tilde{Q}{\left(x^{\left(t\right)},\theta^{\left(t\right)},\varphi^{\left(t\right)}\right)\|}_{2}$$

## 3. Performance Evaluation of Machine Learning in Solving the Inverse Problems

In this section, the performance of predictor functions generated by machine learning is thoroughly investigated. In this study, we adopted ANNs and *k*-NN as methods for machine learning. 

### 3.1. Conditions of Numerical Calculation

**Figure 3:**
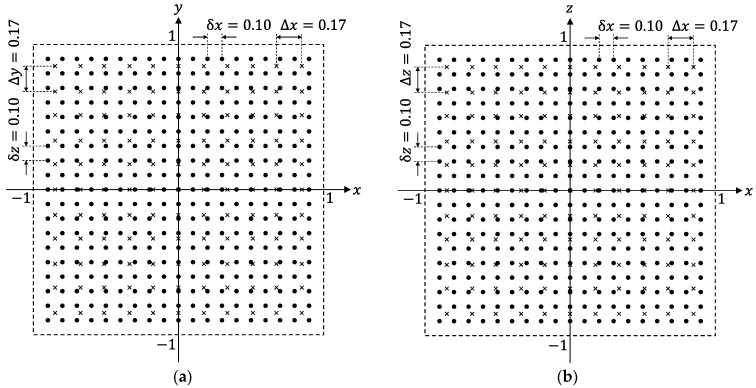
(**a**) *x*-*y* plane and (**b**) *x*-*z* plane sliced in the cubic space shown in Figure 2. The numerical values are described in meters. In this case, the length of one side of the cubic space is supposed to be 2 m. TX positions used to calculate training data are indicated with black dots. The distance between the two nearest dots is 0.10 m. TX positions used to calculate EDF are indicated using crosses. The distance between the two nearest crosses is considered to be 0.17 m. The positions of the dots and crosses do not coincide except for the coordinate origin.

The scenarios and conditions of our investigation are shown in Figure 1, Figure 2 and Figure 3. In this study, we assume that the target (TX) exists inside a cubic space of length (2 m) × height (2 m) × width (2 m). However, the dimensions of the cubic space are not fixed since the scaling law holds in localization with artificially generated magnetic fields. Therefore, the results obtained with the 2 m × 2 m × 2 m cubic space can be easily extended to cubic spaces with arbitrary dimensions. For example, it is possible to obtain a predictor function valid for a cubic space of length (4 m) × height (4 m) × width (4 m) by using the same model without increasing training data. Details on this topic are discussed in [18].

What we should do first is to generate sufficient numbers of training samples in the forms of Equations (10) or (15). Fortunately, we do not have to gather training samples by cumbersome measurement in real systems. It is possible to obtain numbers of training samples just by calculating $B^{\left(k\right)}$ for many different TX-state parameters using Equation (5). The TX-positions chosen to calculate the training samples are indicated by black dots in Figure 3a,b, depicting the *x*-*y* and *x*-*z* planes of the cubic space, respectively. The numerical values in Figure 3 are in meter. Hence, the distance between the two adjacent dots is δx=δy=δz=0.10 m. In this condition, the amount of TX positions for the training samples reaches $19^{3}=6859$. It should be noted that $B^{\left(k\right)}$ is dependent on both positions and angles of the TX. Hence, we must calculate $B^{\left(k\right)}$ for various TX-angle sets $\left(\theta^{\left(t\right)},\varphi^{\left(t\right)}\right)$ for each TX position. Here, we adopt $\delta \theta^{\left(t\right)}=\delta \varphi^{\left(t\right)}=11.25{}^{\circ}$ to calculate the training samples for different TX-angle sets. Therefore, the number of angle sets was 482. Hence, the total number of TX states used for training data reached $N_{\mathrm{training}}=6859\times 482=3,306,038$.

In this study, Wolfram Mathematica 12.0 was used to execute machine learning [18,19]. Mathematica includes highly automated functions related to machine learning. We used the “Predict” command to generate predictor functions P and Q. The command can be executed to automatically generate the predictor functions by inputting training data. We also selected “Quality” as an option command of Mathematica for setting a performance goal. Additionally, it is possible to select several algorithms for machine learning using Mathematica. In this study, we selected “Neural Network” and “Nearest Neighbors” for the algorithms and used a standard computer with Intel Xeon W-2223 and 64-GB RAM.

To evaluate generalization performances of predictor functions obtained by machine learning, we must calculate the EDF values (d) for various TX-state parameters that were not used for calculating training samples. The TX positions used for evaluating the generalization performances are indicated by crosses in Figure 3. It can be confirmed that the positions of the crosses do not coincide with those of dots, which are used for training.

### 3.2. Performance Evaluation of Predictor Functions

We calculated the EDF values and plotted them within *x*-*y* planes for three different TX-state parameters to compare the performances of the predictor functions generated by *k*-NN and ANNs, as shown below.

(21) $$\left(z^{\left(t\right)},\theta^{\left(t\right)},\phi^{\left(t\right)}\right)=\left(0\;m,0{}^{\circ},0{}^{\circ}\right),\left(0.34\;m,30{}^{\circ},30{}^{\circ}\right),\left(0.68\;m,60{}^{\circ},60{}^{\circ}\right)$$

**Figure 4:**
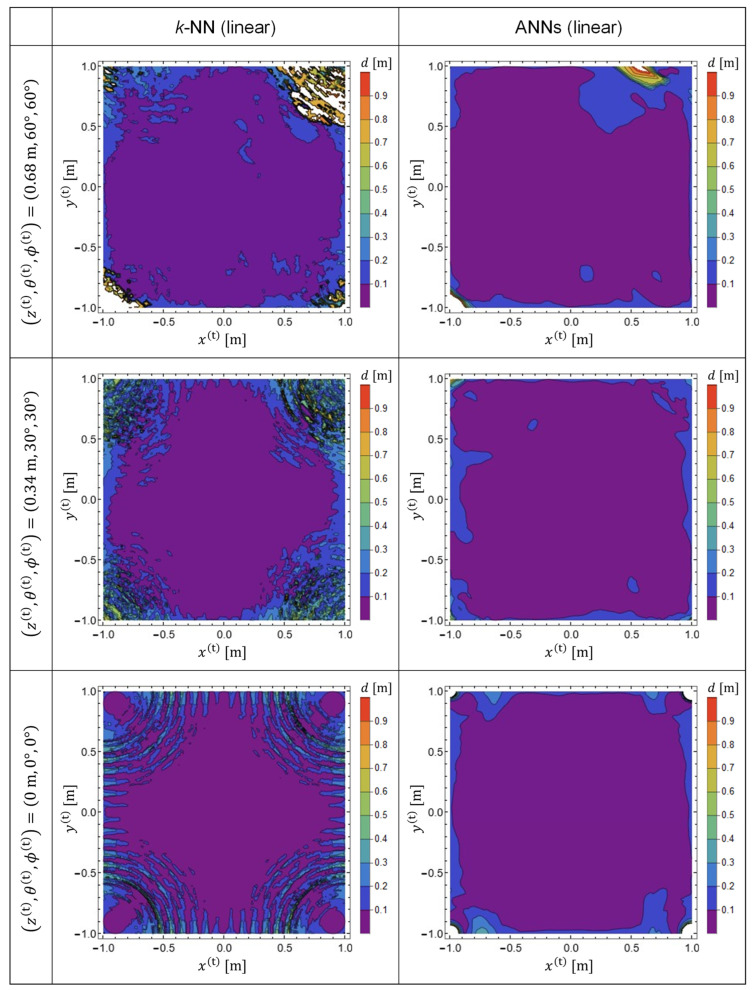
EDF patterns plotted in several cross sections parallel to the *x*-*y* plane. The left and right columns demonstrate results obtained with predictor functions generated by *k*-NN and ANNs, respectively. Both predictor functions (**P**) were generated from training data in linear representation.

The results are presented in Figure 4. The predictor functions used for plotting Figure 4 are P, which were generated from the training data in a linear representation. Note that the TX-state parameters in Equation (21) were not used for the calculation of the training data. Therefore, the results plotted in Figure 4 exhibit the generalization performance of the predictor functions generated by machine learning. The left and right columns indicate the EDF values obtained using *k*-NN and ANNs, respectively. It was observed that, with *k*-NN, the prediction accuracy is reduced in the vicinity of the four corners of the target space [18,19]. Meanwhile, it was confirmed that the prediction accuracy was significantly improved by adopting ANNs.

**Figure 5:**
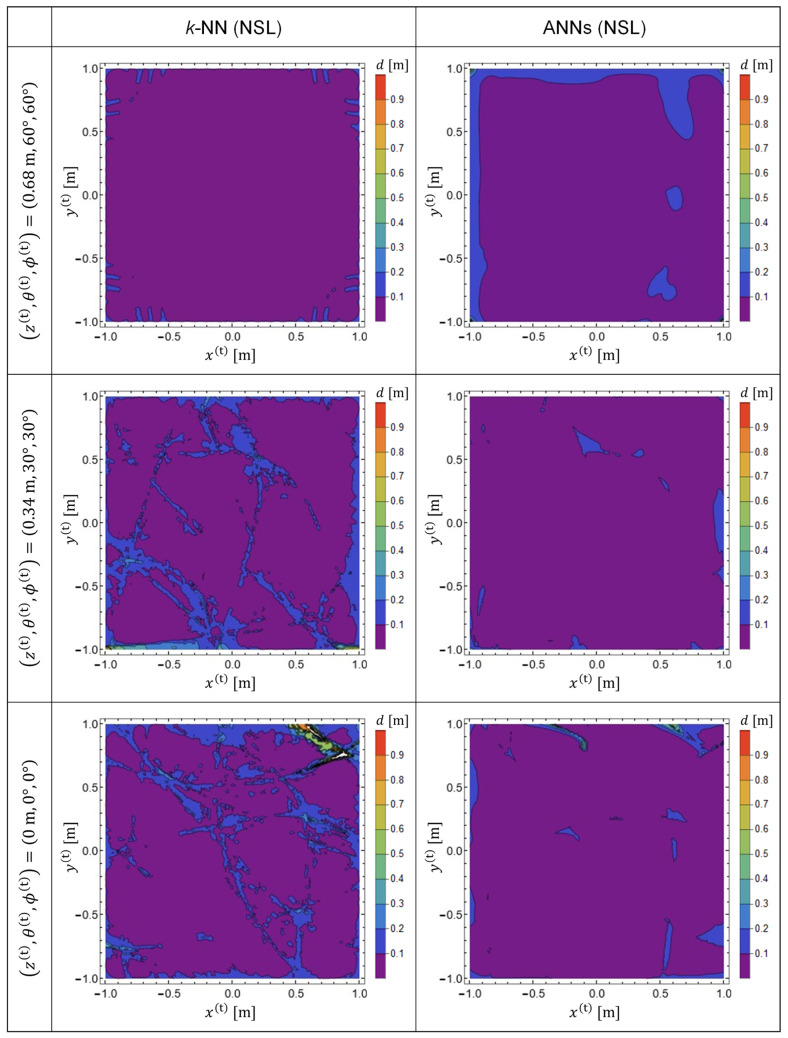
EDF patterns plotted in several cross sections parallel to the *x*-*y* plane. The left and right columns demonstrate results obtained with predictor functions generated by *k*-NN and ANNs, respectively. Both predictor functions (**Q**) were generated from training data in NSL representation.

Figure 5 shows the EDF patterns obtained with the predictor functions Q, which were generated from training data in the NSL representation. By comparing Figure 4 and Figure 5, it is evident that the prediction accuracy is improved for both ANNs and for *k*-NN by using training data in NSL representation. However, it was observed that ANNs showed better performance for both representations of the training data.

Although it has been demonstrated in Figure 4 and Figure 5 that ANNs and NSL representation show better performances, quantitative assessments of the predictor functions have not yet been carried out. Hence, we quantitatively evaluated the performances of the predictor functions via a statistical approach. To do this, we calculated the EDF values at TX positions indicated by crosses in Figure 3 and analyzed the statistical distribution of the values for predictor functions generated from different combinations of the learning algorithms and training-data representations. As shown in Figure 3, the distance between the two adjacent crosses was 0.17 m (Δx=Δy=Δz=0.17 m). The EDF values were calculated for various angles ($\Delta \theta^{\left(t\right)}=\Delta \varphi^{\left(t\right)}=30{}^{\circ}$) for each cross point. As a result, the number of TX states used for the quantitative evaluation of the predictor functions reached 82,522. Since the TX states used to generate the training samples were not included in the 82,522 states, the statistical distribution of the EDF values reflects generalization performances of the predictor functions.

**Figure 6:**
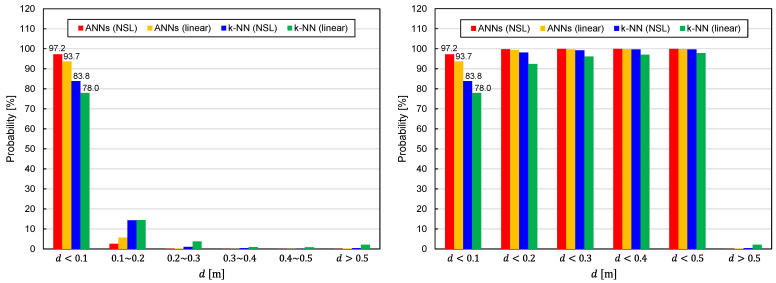
Statistical distributions of EDF values obtained using four different combinations of the learning algorithms (ANNs/*k*-NN) and training-data representations (NSL/linear).

**Figure 7:**
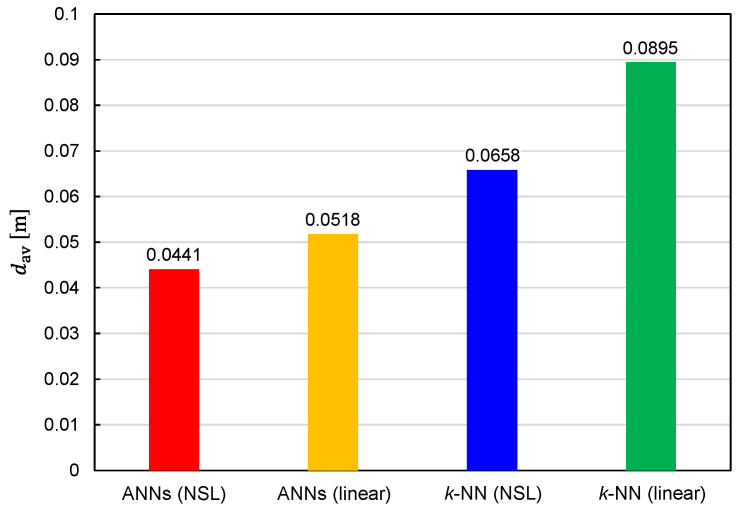
Average EDF values obtained using four different combinations of the learning algorithms (ANNs/*k*-NN) and training-data representations (NSL/linear).

The statistical distributions of the EDF values are shown in Figure 6. It can be seen that the predictor functions generated by ANNs exhibit better performance for both representations of the training data (NSL and linear). With regard to the training-data representations, the NSL representation is superior to the linear representation, regardless of the learning algorithms. Figure 7 illustrates the EDF values averaged over 82,522 TX states. The average EDF value is denoted by $d_{\mathrm{av}}$. The benefits of using ANNs and NSL representation are again confirmed by Figure 7.

**Figure 8:**
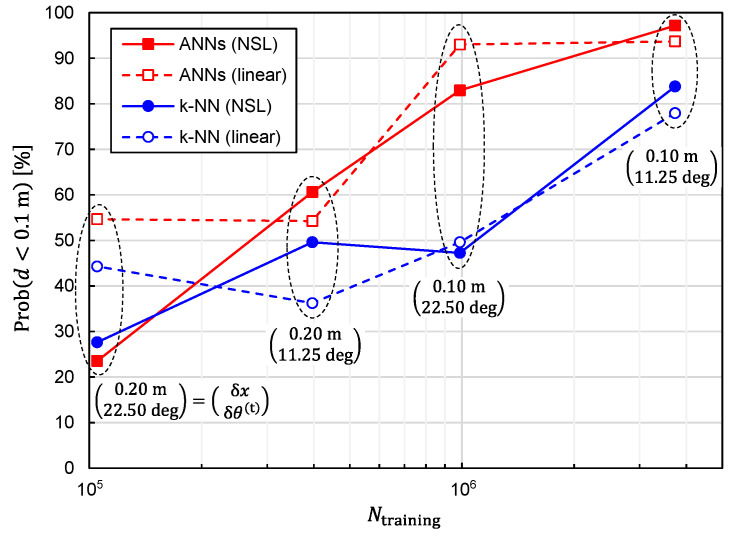
Relationship between Prob(d<0.1 m) and the number of training samples plotted for predictor functions generated from four different combinations of the learning algorithms (ANNs/*k*-NN) and training-data representations (NSL/linear).

**Figure 9:**
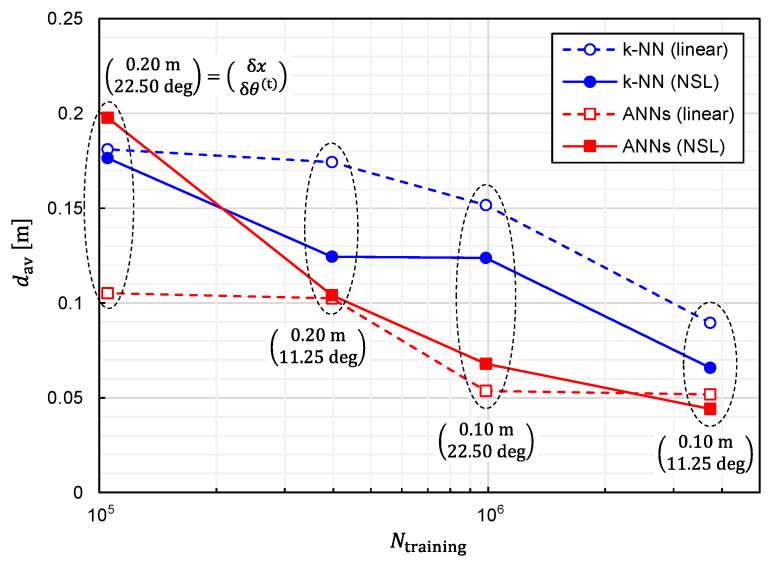
Relationship between average EDF values and the number of training samples plotted for predictor functions generated from four different combinations of the learning algorithms (ANNs/*k*-NN) and training-data representations (NSL/linear).

It is also critical to investigate the relationship between the prediction accuracy and the number of training samples, $N_{\mathrm{training}}$. We plotted Prob(d<0.1 m) and $d_{\mathrm{av}}$ as functions of $N_{\mathrm{training}}$ in Figure 8 and Figure 9, respectively, to investigate the relationship. Since reducing $N_{\mathrm{training}}$ is equivalent to reducing δx(=δy=δz) and $\delta \theta^{\left(t\right)}\left(=\delta \varphi^{\left(t\right)}\right)$, the simulations have been executed for different combinations of $\left(\delta x,\delta \theta^{\left(t\right)}\right)$. It can be observed from these figures that the prediction accuracy was improved by increasing $N_{\mathrm{training}}$. Moreover, it is expected that the prediction accuracy will be further improved by increasing $N_{\mathrm{training}}$ except for the combinatorial use of ANNs and linear representation. In other words, the situation of overfitting has not yet occurred for $N_{\mathrm{training}}=3,306,038$.

In real-world applications, signals detected by sensors are affected by noise and irregularities of fabricated coils. Therefore, it is important to estimate the influences of these factors on prediction accuracy. Here we first discuss the influences of noise.

Since the amplitude of received signals depends on TX-state parameters, it is valid to define the reference magnetic-field amplitude for quantitatively discussing the influences of noise. Thus, we introduce the reference magnetic-field amplitude defined by

(22) $$B_{\mathrm{ref}}≜\left|B_{z}^{\left(k\right)}\left(0,0,0,0,0\right)\right|.$$

It is understood that $B_{\mathrm{ref}}$ means the magnetic-field amplitude generated at the sensor positions by the TX directed toward *z*-axis at a coordinate origin. Note that $B_{\mathrm{ref}}$ is independent of *k* when the sensors are located at the corners of a cubic space as shown in Figure 2. We also define the signal-to-noise ratio (SNR) of sensors as

(23) $$\mathrm{SNR}\;\left[\mathrm{dB}\right]≜20{\;\log}_{10}\left(\frac{B_{\mathrm{ref}}}{B_{\mathrm{ein}}}\right),$$

where $B_{\mathrm{ein}}$ denotes amplitude of equivalent-input noise associated with each coil of the sensor. By considering the noise, the signal amplitude detected by the sensor can be written as

(24) $$B_{i}^{\left(k\right),\mathrm{real}}=B_{i}^{\left(k\right)}\sqrt{1+{\left(\frac{B_{\mathrm{ein}}}{B_{i}^{\left(k\right)}}\right)}^{2}}=B_{i}^{\left(k\right)}\sqrt{1+10^{-\frac{\mathrm{SNR}\;\left[\mathrm{dB}\right]}{10}}\cdot {\left(\frac{B_{\mathrm{ref}}}{B_{i}^{\left(k\right)}}\right)}^{2}},\;$$

where $B_{i}^{\left(k\right),\mathrm{real}}$ and $B_{i}^{\left(k\right)}$ denote the amplitudes of detected signals with and without noise, respectively. 

**Figure 10:**
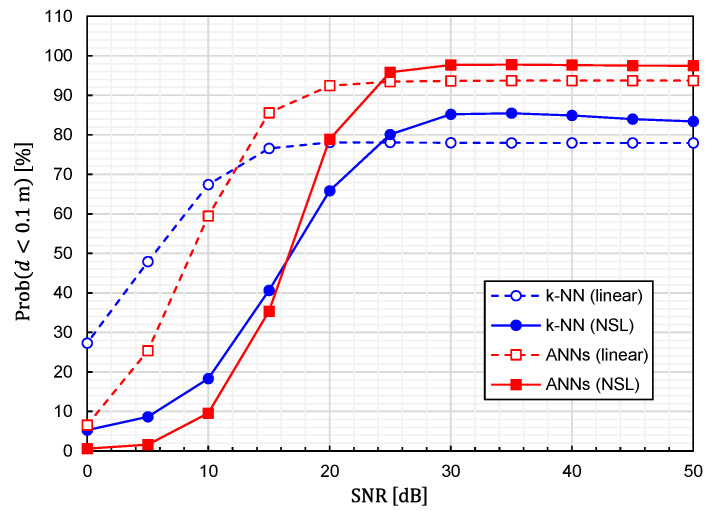
Relationship between Prob(d<0.1 m) and SNR for predictor functions generated from four different combinations of the learning algorithms (ANNs/*k*-NN) and training-data representations (NSL/linear).

**Figure 11:**
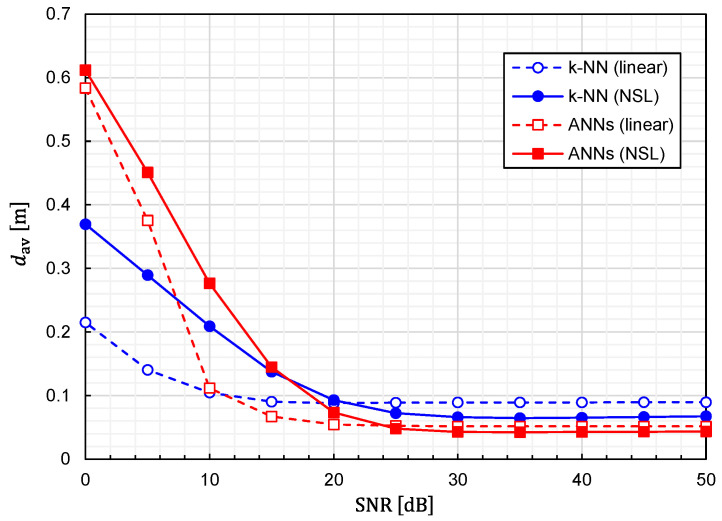
Relationship between average EDF values and SNR for predictor functions generated from four different combinations of the learning algorithms (ANNs/*k*-NN) and training-data representations (NSL/linear).

Using Equation (24), we calculated the prediction accuracy as functions of the SNR. The results for Prob(d<0.1 m) and $d_{\mathrm{av}}$ are plotted in Figure 10 and Figure 11, respectively. It is observed that noise immunity depends on training-data representations. For NSL representation, the prediction accuracy is almost constant for SNR>25 dB. On the other hand, for linear representation, it is kept constant for SNR>15 dB. It is interesting to see that linear representation is superior to NSL representation in terms of noise immunity.

**Figure 12:**
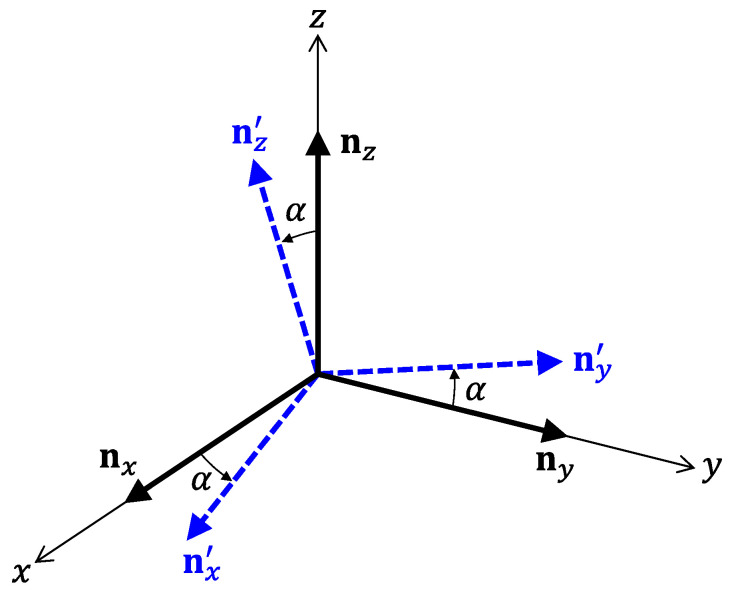
Relationship between coordinate axes and unit vectors perpendicular to receiving coils. Ideal vectors are denoted by $n_{x}$, $n_{y}$ and $n_{z}$. On the other hand, $n_{x}^{\prime }$, $n_{y}^{\prime }$, and $n_{z}^{\prime }$ represent the unit vectors that include angle errors.

Next, we consider the influences of misalignment of receiving coils. In this study, we suppose that each sensor is equipped with the three coils. Let us denote unit vectors perpendicular to the three coils by $n_{x}$, $n_{y}$, and $n_{z}$. It is obvious that $n_{x}$, $n_{y}$, and $n_{z}$ must be parallel to *x*, *y*, and *z*-axes fixed to a target space, respectively. However, it is difficult to align the coils so that $n_{x}$, $n_{y}$, and $n_{z}$ become completely parallel to the coordinate axes. As shown in Figure 12, we consider the situation of the misalignment, where the wrongly directing unit vectors are denoted by $n_{x}^{\prime }$, $n_{y}^{\prime }$, and $n_{z}^{\prime }$. For simplicity, let the error angle α be common for all unit vectors. Moreover, we assume that $n_{x}^{\prime }$, $n_{y}^{\prime }$, and $n_{z}^{\prime }$ are parallel to *x*-*y*, *y*-*z*, and *z*-*x* planes, respectively. In this situation, the influences of the misalignment can be written as

(25) $$\left(\begin{array}{c} B_{x}^{\left(k\right),\mathrm{ma}}\\ B_{y}^{\left(k\right),\mathrm{ma}}\\ B_{z}^{\left(k\right),\mathrm{ma}} \end{array}\right)=\left(\begin{array}{ccc} \cos\alpha & \sin\alpha & 0\\ 0 & \cos\alpha & \sin\alpha \\ \sin\alpha & 0 & \cos\alpha \end{array}\right)\left(\begin{array}{c} B_{x}^{\left(k\right)}\\ B_{y}^{\left(k\right)}\\ B_{z}^{\left(k\right)} \end{array}\right),$$

where $B_{i}^{\left(k\right),\mathrm{ma}}$ and $B_{i}^{\left(k\right)}$ denote amplitudes of the detected signals with and without the misalignment, respectively.

**Figure 13:**
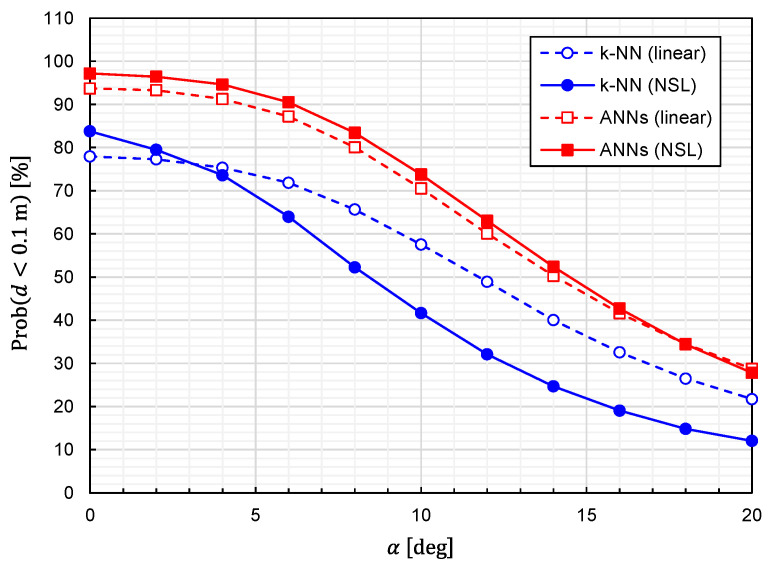
Relationship between Prob(d<0.1 m) and error angle for predictor functions generated from four different combinations of the learning algorithms (ANNs/*k*-NN) and training-data representations (NSL/linear).

**Figure 14:**
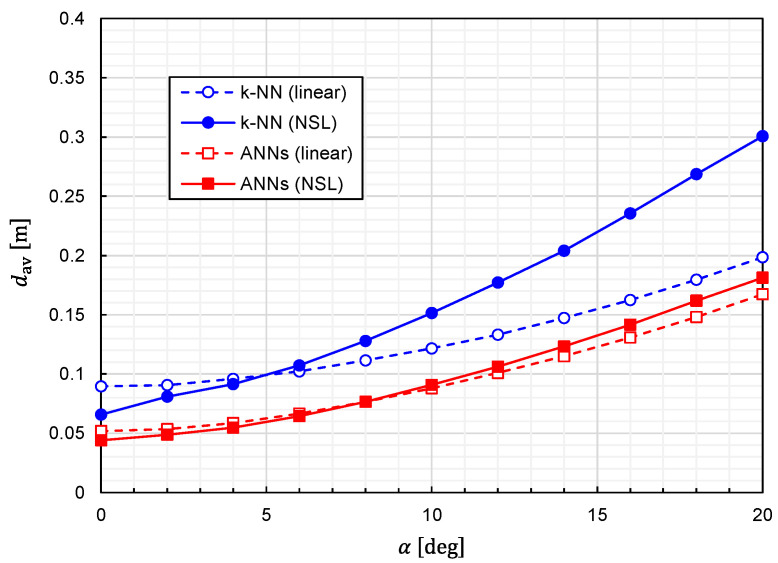
Relationship between average EDF values and error angle for predictor functions generated from four different combinations of the learning algorithms (ANNs/*k*-NN) and training-data representations (NSL/linear).

Using Equation (25), we calculated the prediction accuracy as functions of α. The results for Prob(d<0.1 m) and $d_{\mathrm{av}}$ are plotted in Figure 13 and Figure 14, respectively. It is observed that the predictor function generated from the combination of *k*-NN and NSL is the most sensitive to α. The other three predictor functions show similar characteristics against α. We can say that the influences of the misalignment are limited for α<5 deg.

**Figure 15:**
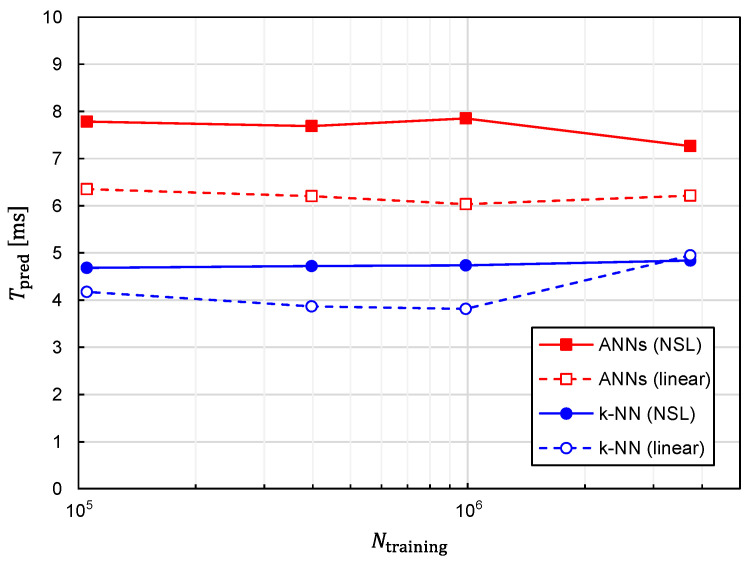
Relationship between the time required for predicting a TX position and the number of training samples plotted for predictor functions generated from four different combinations of the learning algorithms (ANNs/*k*-NN) and training-data representations (NSL/linear).

In addition to prediction accuracy, the computational speed is another critical aspect that needs to be evaluated. Since one of the advantages of machine learning to the conventional optimization method is the speed of predicting target positions, it is imperative to evaluate the time required for the prediction with machine learning, which we denote as $T_{\mathrm{pred}}$. The relationship between $T_{\mathrm{pred}}$ and $N_{\mathrm{training}}$ is plotted in Figure 15. The following three features can be observed in Figure 15.

1. $T_{\mathrm{pred}}$ is almost independent of $N_{\mathrm{training}}$;
2. $T_{\mathrm{pred}}$ is increased by approximately 1.5 times with ANNs in comparison with *k*-NN;
3. $T_{\mathrm{pred}}$ is increased by approximately 1.2 times with training data of NSL representation in comparison with those of linear representation.

In terms of prediction accuracy, the best combination of the algorithm and training-data representation is that of the ANNs and NSL. Fortunately, $T_{\mathrm{pred}}$ considered with the best combination is kept less than 10 ms and is not drastically increased in comparison with the other combinations. Therefore, real-time tracking is possible with the best combination.

**Figure 16:**
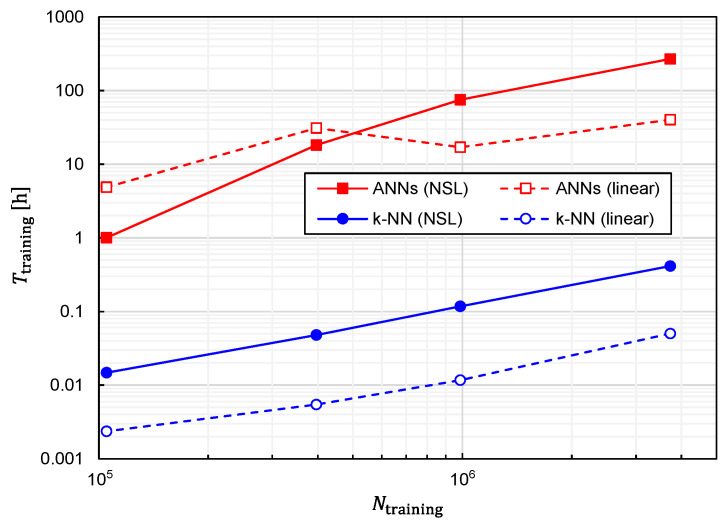
Relationship between the time required for training and the number of training samples plotted for predictor functions generated from four different combinations of the learning algorithms (ANNs/*k*-NN) and training-data representations (NSL/linear).

Regarding the computational speed, another critical index is the time required to generate a predictor function from training data, which we denote as $T_{\mathrm{training}}$. Therefore, we also investigated the relationship between $T_{\mathrm{training}}$ and $N_{\mathrm{training}}$, which is plotted in Figure 16. The following three features can be observed in Figure 16.

1. $T_{\mathrm{training}}$ is an almost monotonically increasing function of $N_{\mathrm{training}}$;
2. For large $N_{\mathrm{training}}$, $T_{\mathrm{training}}$ is increased by approximately 500 to 1000 times with ANNs in comparison with *k*-NN;
3. For large $N_{\mathrm{training}}$, $T_{\mathrm{training}}$ is increased by approximately 5 to 10 times with training data of NSL representation in comparison with those of linear representation.

From a practical viewpoint, the only drawback of the best combination is that $T_{\mathrm{training}}$ becomes considerably longer. In fact, it takes 269 h for $N_{\mathrm{training}}=3,306,038$ with the computer used in this study (Intel Xeon W-2223 and 64-GB RAM). The considerably large $T_{\mathrm{training}}$ is primarily due to the adoption of ANNs. However, as shown in Figure 8 and Figure 9, a better prediction accuracy is obtained with ANNs. Therefore, there is a trade-off relationship between $T_{\mathrm{training}}$ and prediction accuracy. The performances obtained thus far are summarized in Table 1.

**Figure 17:**
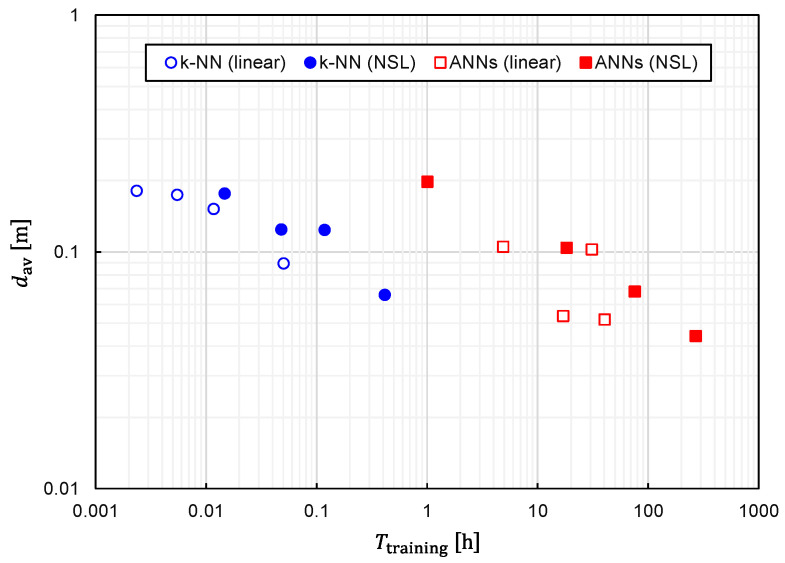
Relationship between the average EDF values and time required for training plotted for several different numbers of training samples.

We plotted the relationship between $d_{\mathrm{av}}$ and $T_{\mathrm{training}}$ for several different values of $N_{\mathrm{training}}$ in Figure 17 to clearly establish the trade-off relationship. As expected, the trade-off relationship can be seen as a whole. However, it was also observed that *k*-NN and ANNs formed different clusters. The right cluster composed of squares suggests that ANNs are suitable for obtaining the best prediction accuracy, although $T_{\mathrm{training}}$ becomes considerably long. Meanwhile, the left cluster composed of circles implies that a fairly good prediction accuracy is obtainable with *k*-NN while keeping $T_{\mathrm{training}}$ fairly short.

## 4. Conclusions

Localization systems that utilize artificially generated magnetic fields are required to calculate target positions based on the information of magnetic fields detected by multiple sensors. This calculation is not easy since it involves solving nonlinear inverse problems. In this study, we demonstrated that machine learning is suitable for solving inverse problems.

Our emphasis was on comparing performances obtained with *k*-NN and ANNs that were adopted for machine learning. We numerically evaluated the accuracy of the target positions predicted by *k*-NN and ANNs by considering the 2 m × 2 m × 2 m cubic space. Prob(d<0.1 m) and $d_{\mathrm{av}}$ obtained with *k*-NN were 84% and 66 mm, respectively, and those obtained with ANNs were 97% and 44 mm, respectively. It was demonstrated that ANNs are superior to *k*-NN in terms of prediction accuracy.

Furthermore, we also evaluated the computational times taken with the *k*-NN and ANNs. Accordingly, $T_{\mathrm{pred}}$ and $T_{\mathrm{training}}$ obtained with *k*-NN were 4.8 ms and 0.4 h and those obtained with ANNs were 6.2 ms and 269 h, respectively. It was demonstrated that $T_{\mathrm{pred}}$ obtained with both *k*-NN and ANNs was short enough to execute real-time tracking. However, it was revealed that ANNs require much longer $T_{\mathrm{training}}$ than *k*-NN. Therefore, *k*-NN remains a valid method for generating fairly good predictor functions within limited training times.

## Figures and Tables

**Table 1:** Summary of performances of predictor functions generated from four different combinations of the learning algorithms (ANNs/*k*-NN) and training-data representations (NSL/linear).

	ANN (NSL)	ANN (Linear)	*k*-NN (NSL)	*k*-NN (Linear)
Prob(d<0.1 m) [%]	97.2	93.7	83.8	78.0
$d_{\mathrm{av}}\;\left[\mathrm{mm}\right]$	44.1	51.8	65.8	89.5
$T_{\mathrm{pred}}\;\left[\mathrm{ms}\right]$	7.27	6.21	4.84	4.95
$T_{\mathrm{training}}\;\left[h\right]$	269	40.3	0.414	0.0503
